# Carbon Dioxide Monitoring inside an Australian Brewery Using an Internet-of-Things Sensor Network

**DOI:** 10.3390/s22249752

**Published:** 2022-12-13

**Authors:** Amer Hawchar, Solomon Ould, Nick S. Bennett

**Affiliations:** 1 Centre for Advanced Manufacturing, University of Technology Sydney, Broadway, Ultimo, NSW 2007, Australia; 2 Radio Frequency and Communication Technologies Laboratory, University of Technology Sydney, Broadway, Ultimo, NSW 2007, Australia

**Keywords:** CO_2_, Internet of Things, sensor, indoor air quality

## Abstract

Maintaining a high standard of indoor air quality (IAQ) is vital to ensuring good human health. The concentration of CO_2_ in air is a good proxy for IAQ, while high levels of CO_2_ have been shown to cause cognitive or physiological impairment. Work environments that generate CO_2_ as an inherent part of their business present a unique and significant risk in terms of poor IAQ. Craft breweries generate CO_2_ and, unlike larger breweries, often lack the technology to capture and re-use the fermentation CO_2_ for beer carbonation. The purpose of this study is to demonstrate that the venting of fermentation CO_2_ and the unintentional venting of CO_2_ during the filling of CO_2_ storage tanks can cause the indoor CO_2_ levels to rise significantly. This is shown by monitoring CO_2_ levels inside an Australian craft brewery using a newly developed system containing three Internet of Things (IoT) sensor nodes positioned strategically in different sections of the brewery. The maximum CO_2_ level recorded was in excess of 18,000 ppm, with the maximum time period levels exceeding 1000 and 10,000 ppm being equivalent to 425 and 26 min, respectively. The identification of differences in measured CO_2_ at different times and locations throughout the brewery reveals that a single hard-wired CO_2_ sensor may be inadequate to support IAQ monitoring. For this purpose, a network of portable or wearable CO_2_ sensor nodes may be most suitable. The battery life of the sensors is a key consideration, and the current sensor battery life is too short. Low-power sensors and communication protocols are recommended for this task.

## 1. Introduction

The Environmental Protection Agency (EPA) regulates indoor and outdoor air quality in the United States. They have stated that indoor pollutant levels exceed 100 times higher than their outdoor counterparts and have ranked poor air quality in the top 5 environmental risks to public health [1]. It is important to ensure that indoor air quality (IAQ) is free of elevated levels of pollutants that may cause long/short term injury, and this applies both to domestic residences as well as indoor workplaces. The risks associated with poor IAQ are equally relevant across the globe; however, the Australian Building Codes Board (ABCB) states “There are no specific legislated standards for IAQ in Australia, although there are exposure standards set for a range of chemicals in industrial environments” [2].

### 1.1. Air Pollutants

The main pollutants with regard to IAQ are particle matter (PM), volatile organic compounds (VOCs), nitrous oxides (NO_X_), ozone (O_3_), sulfur dioxide (SO_2_), and carbon oxides (CO and CO_2_). Other pollutants include heavy metals, aerosols, radon (Rn), pesticides, biological allergens and microorganisms [3].

PM is defined as fine carbonaceous particles that are associated with absorbed organic chemicals and reactive metals [3]. PM is caused by particles that migrate from outdoor environments, or particles generated indoors via cooking, combustion of fossil fuels, smoking, or machine operation [3]. PM of diameter <0.1 μm is of extreme concern as it can be inhaled deep into the lung’s alveoli, which can affect the lungs and heart, potentially leading to serious health injuries [3,4,5].

VOCs are gaseous organic compounds that contain chemicals emitted from liquids or solids [6], and formaldehyde is the most common [3]. VOCs are released from cooking, smoking, use of cleaning or personal care products, indoor chemical reactions, penetration of outdoor air, and building materials [7,8,9,10,11]. VOCs can affect humans via inhalation, ingestion, or dermal contact [3]. Long-term exposure can potentially cause cancer [12].

NO_X_ consists of nitric oxide (NO) and nitrogen dioxide (NO_2_) gases, commonly caused by combustion sources [13]. Elevated levels of NO_X_ can cause increased asthmatic reactions and respiratory damage [13].

Ozone is produced by the photochemical reactions of atmospheric O_2_, NO_x_, and VOCs [3]. Ozone reacts with indoor pollutants, which can create products that irritate humans, via inhalation or skin exposure, and damage materials [3,14]. Ozone indoors emerges mainly from electrical devices and penetration of the outdoor air [15].

Sulfur dioxide is a gas that is most commonly produced by the combustion of fossil fuels and can combine with aerosols or PMs [16]. SO_2_ is emitted indoors by vented gas appliances, oil furnaces, tobacco smoke, kerosene heaters, and coal or wood stoves [17]; however, it can also enter indoor spaces via penetration from outdoor air [18]. The hourly concentration of SO_2_ indoors is often below 20 ppb [19]. Exposure to SO_2_ can impair respiratory function via inhalation [3].

CO_X_ consists of carbon monoxide (CO) gas and carbon dioxide (CO_2_) gas. CO gas is produced indoors by combustion processes; however, it can also enter indoor spaces via the penetration of outdoor air [20]. The average concentration of CO indoors without a combustion source is equal to 0.5–5 ppm; however, when a gas stove is added, CO levels can rise higher than 30 ppm [3]. Exposure to low concentrations of CO can impact cardiovascular and neurobehavioral processes, while high concentrations can cause unconsciousness or death [21].

### 1.2. Carbon Dioxide as a Pollutant

CO_2_ is a colorless and odorless gas that exists in the atmosphere at levels of approximately 400 ppm. CO_2_ is often produced indoors by the human respiratory system, combustion of fossil fuels and fermentation. In recent years, the CO_2_ level indoors has been used as a proxy for the assessment of IAQ [22,23,24]. Cognitive performance is impacted by CO_2_ levels exceeding 1000 ppm, which includes decision making and problem resolution [25,26,27,28], while CO_2_ levels exceeding 10,000 ppm results in impaired physiological effects such as increased respiratory rate, respiratory acidosis, metabolic stress (decreased blood calcium or urine phosphorus), increased brain blood flow, and increased minute ventilation [29,30,31,32].

Work environments that generate CO_2_ as an inherent part of their business present a unique and significant risk in terms of IAQ. An example is breweries, who generate CO_2_ as a by-product of fermentation, where yeast turns glucose into alcohol and CO_2_, something that is a critical part of beer production. For relatively large breweries, technology exists to capture and re-use the fermentation CO_2_ for beer carbonation; however, this technology is inaccessible to smaller breweries due to its high costs. In the case of small or medium-sized breweries, CO_2_ is normally vented from the fermentation tanks directly to the indoor environment. CO_2_ for carbonation is usually purchased separately and is directly pumped into large indoor/outdoor storage tanks via delivery trucks. The venting from fermentation and the unintentional venting from CO_2_ storage tank refilling presents a risk that could cause indoor CO_2_ levels to exceed safe limits, and therefore adequate CO_2_ monitoring systems must be present.

### 1.3. Carbon Dioxide Monitoring

**Figure 1:**
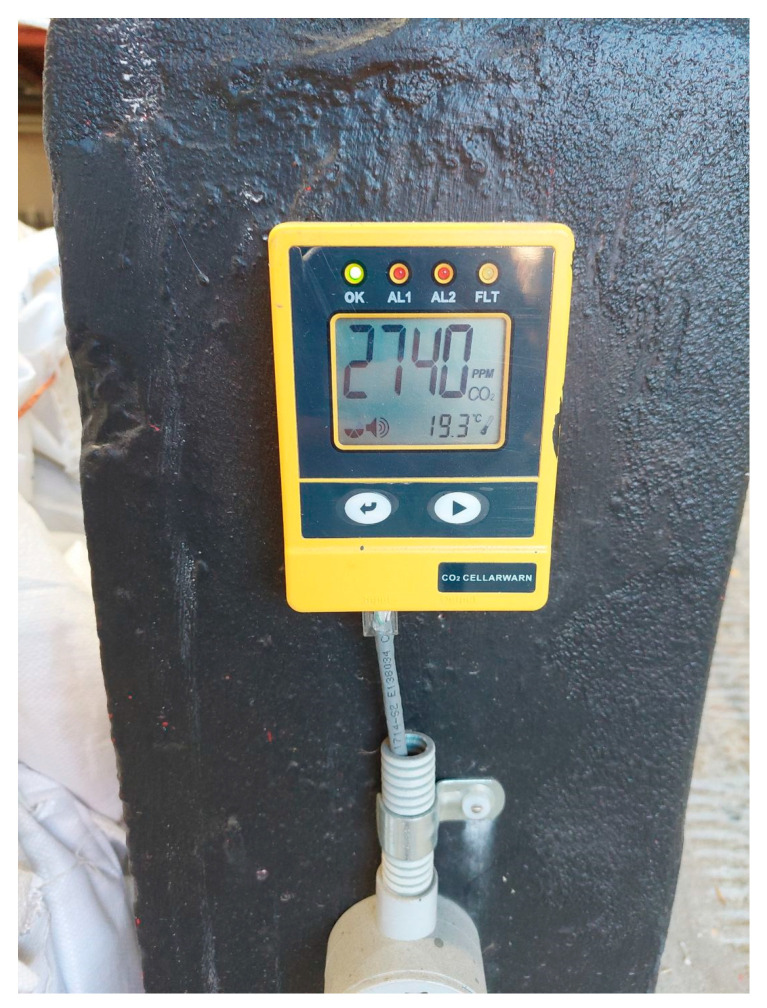
An example of a hard-wired CO_2_ monitoring system used in an Australian brewery.

Craft breweries in Australia typically use a single hard-wired CO_2_ monitoring system (Figure 1) to track IAQ and prevent possible health effects. Such alarms trigger when extremely high levels of between 15,000 and 30,000 ppm are detected. This limits the effectiveness of the system in two ways: Firstly, as the sensor is fixed, it cannot monitor individual areas of the workplace. This may put workers at risk who are in unmonitored areas. Secondly, the alarm only triggers at extremely high levels; therefore, the workers may be regularly at risk of low-level CO_2_ side-effects such as decreased cognitive performance without realizing. Furthermore, since brewery workers are consistently engaged in manual tasks, e.g., using forklifts, lifting heavy products, and hosing down equipment, which may leave the floor wet, there is an increased risk of work-related injuries occurring.

In recent years, research has been conducted to improve the aforementioned systems by including more sensitive sensors, Internet of Things (IoT) connectivity and improved data collection and dashboarding software.

In reference [33], Tran et al. (2017) developed a battery-free sensor that is capable of monitoring IAQ in real-time, though not specifically CO_2._ The sensor consisted of three main components, an entirely passive ultra-high frequency (UHF) smart tag for communication with a UHF radio frequency identification (RFID) reader, a smart sensing module with ultra-low power sensors and a microcontroller unit (MCU), and a radio frequency (RF) energy harvester. The sensor system measured the concentration of volatile organic compounds, ambient temperature, relative humidity, and atmospheric pressure. The sensing circuit that was designed using ultra-low power sensors and a microcontroller unit (MCU) consumed only 0.5 mW.

In references [34,35,36,37], Marques et al. (2019), Kim et al. (2014), Pitarma et al. (2017), and Abraham et al. (2014) developed systems that used IoT architecture for monitoring IAQ using microsensors for data acquisition and open-source technology for both processing and data transmission, while allowing the data collected to be accessed, in real time, from various sites simultaneously though the web and/or mobile applications.

In reference [34], Marques et al. (2019) developed the iAirCO_2_ system, a method that real-time monitored CO_2_ levels using IoT. The iAirCO_2_ was composed of a hardware prototype for ambient data collection, web/smartphone software for data collection via Wi-Fi, and an SQL Server. The primary reason was to enhance living environments by providing IAQ data that can be accessed by doctors in order to support medical diagnostics.

Kim et al. (2014) [35] developed a system that monitored particulate matter, ozone, carbon monoxide, carbon dioxide, nitrous oxides, sulfur dioxide, VOCs, temperature, and humidity. The system contained a prototype sensor module that used a Raspberry Pi, while incorporating a smoothing algorithm to prevent temporary sensor errors, and an aggregation algorithm to reduce the network traffic and power consumption. The main focus was to monitor IAQ on a real-time basis in urban ecosystems.

In reference [36], Pitarma et al. (2017) developed a wireless sensor network for the monitoring and collection of IAQ using Arduino, XBee modules/microsensors and ZigBee protocol. Temperature, humidity, carbon monoxide, CO_2_ and luminosity data were monitored and later accessed via an Android application and a web portal. The purpose of this research was to provide an effective indoor air quality assessment to prevent sick building syndrome.

Abraham et al. (2014) [37] developed a simplified ZigBee system for IAQ monitoring applications using the Arduino platform. The system consisted of low-cost CO_2_, VOC, temperature and humidity sensors; however, the system did not provide any mobile computing solution for IAQ evaluation or analytics. The objective was to reduce indoor air pollution to improve public health.

### 1.4. Carbon Dioxide Monitoring inside a Working Brewery

CO_2_ IAQ testing inside craft breweries using IoT technology is relatively new and therefore prior research is limited; however, in reference [38], Huizen (2020) developed a wearable system that used IoT to monitor brewery IAQ, but the system was bulky and relatively difficult to implement due to the inclusion of a Raspberry Pi, camera, power supply and CO_2_ sensor that had to be worn like a backpack at all times.

The purpose of this research was to expand on previous research outlined above by creating a low cost, portable and easy to implement method of monitoring IAQ. The novelty of this work is its focus on ensuring safe working environments inside breweries by monitoring and recording the CO_2_ levels using IoT and open-source technology.

## 2. Methodology

**Figure 2:**
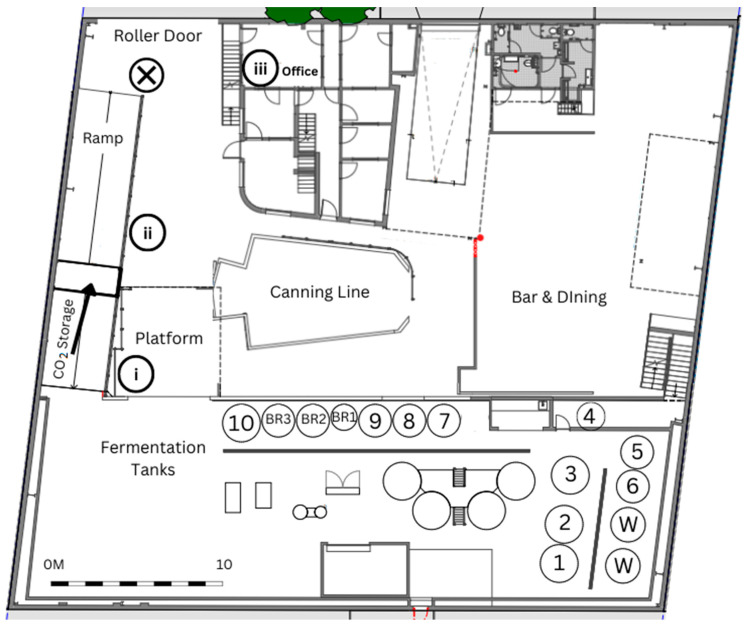
A floorplan of the brewery. Sensor positions, X: hardwired. i: Node 1, ii: Node 2, iii: Node 3, W: water tanks, BR1–3: bright beer tank, numbers 1–10 indicate the fermentation tank numbers, the arrow indicates the CO_2_ storage.

A working brewery in Sydney, Australia was selected as the use case to investigate the dynamic concentration of CO_2_ over the course of various working cycles during a period of two months. The brewery produces approximately 1.3 million liters of beer annually, is approximately 900 m^2^ in area across 2 levels, has 10 fermentation tanks, 3 bright beer tanks, and 2 water tanks, ranging from 4000–12,000 L. As shown in Figure 2, the brewery top floor contains all the fermentation and brewing infrastructure, while the bottom floor contains the office, canning line and bar/dining area. The brewery has some permanent staff supported by a casual workforce of approximately 50 employees, with a maximum of 5 workers working on the top floor, 5 in the canning line, and 3 in the office, at any one time. The main ventilation point of the brewery is the large roller door at the bottom of the ramp and just outside the office. The brewery is equipped with the hard-wired CO_2_ monitoring system shown in Figure 1.

**Figure 3:**
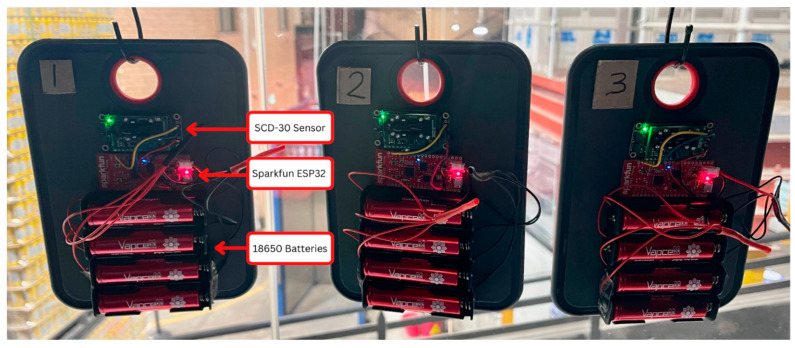
IoT CO_2_ sensor nodes 1–3.

**Figure 4:**
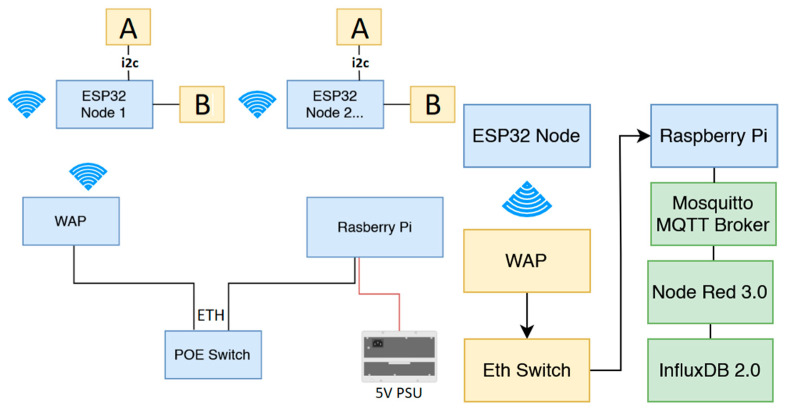
Digital architecture for the sensor network, A: SCD-30 sensor, B: 18650 batteries, WAP: wireless access point, POE: power over ethernet, ETH: ethernet.

The developed IoT CO_2_ monitoring system consisted of the physical sensor nodes (Figure 3) and the backend infrastructure (Figure 4). The sensor portion consisted of four lithium ion 18650 batteries (14,000 mAh total) connected in series to a Sparkfun ESP32 Thing board, which receives and transmits data over Wi-Fi from the Adafruit SCD-30 CO_2_ sensor. The SCD-30 can measure CO_2_ (ppm) in the range of 400 to 10,000 ppm with a resolution of ± (30 ppm + 3%) ppm [39]. It can also measure air temperature (°C) and humidity (%).

Arduino IDE was used to program and connect the ESP32 boards to the Wi-Fi Access Point, which was the primary network connection. Once connected to the local Wi-Fi network, the sensor nodes were then connected to a Mosquito MQTT Broker instance running on a Raspberry PI under Rasbian OS. The nodes uploaded CO_2_ data every 30 s by publishing to the broker. Running simultaneously on the Raspberry PI were instances of Node-Red 3.0 and Influx DB 2.0. Node-Red was used to subscribe to the MQTT topics and add any extra tags required to the data before it was written to Influx DB. Each node was given a unique field name in the database and tags were added as needed to provide metadata. Influx DB 2.0 allowed the use of the new graphical user interface previously named Chronograf to integrate the data and find patterns in the CO_2_ levels.

Three identical IoT monitoring sensor nodes were assembled to monitor the CO_2_ levels in three different sections of the brewery’s workplace. The sensors were firstly placed in close vicinity of a pre-existing CO_2_ monitoring system and calibrated. Node 1 was then placed near the fermentation tanks as this location was where CO_2_ venting occurred, while Node 2 was placed near the canning line as this was directly below the fermentation tanks and was a suspected location for CO_2_ since it is denser than air and might sink to this location once released. It is also in close proximity to the bay where CO_2_ for carbonation is stored. Finally, Node 3 was placed in the office area. Figure 2 depicts the precise location of the nodes and hard-wired alarm at the ground floor and top floor, respectively. An additional manual data stream was created primarily to determine when the brewery was venting CO_2_. A venting sheet was created and printed for the staff to take note whenever venting occurred. The sheet recorded when (date/time) venting occurred, venting duration, the fermentation tank number and any additional comments. The bright beer tanks (BR1-3) are vessels that contain beer that is ready for packaging and that has already been carbonated. In the case of bright beer tank venting, the vent is done after the beer is transferred out of the tank to packaging (canning) and is done to remove excess CO_2_ in the tank. Similarly, when the carbonation CO_2_ storage tank was refilled by a commercial supplier, was also manually recorded.

The influx database was observed at the end of every working day to determine any CO_2_ trends that may have resulted from venting. The following day, the database information was compared to the manual data stream to analyze the impact of the venting and to draw conclusions.

Finally, observation sessions were undertaken in the brewery to record numbers and movements of workers throughout the brewery space.

## 3. Results and Discussion

A 10-day subset of CO_2_ venting events within the brewery is summarized in Table 1. Shown is the approximate venting time—as recorded by the brewery staff—along with the duration of the event. Vents occurred for approximately 30 min, unless otherwise stated. This was the case in all but a few cases.

### 3.1. Maximum CO_2_ Concentrations

**Figure 5:**
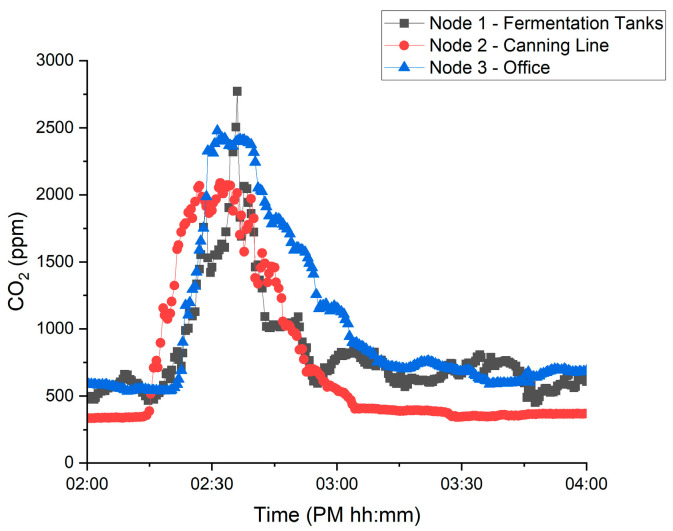
CO_2_ Measured versus time for venting event 5b at 3 measurements locations within the brewery.

Figure 5 contains an example extract from the database for the CO_2_ levels measured in ppm for all three nodes against time for the venting event 5b. The baseline CO_2_ levels within the brewery workplace prior to venting reside at between 400–700 ppm—values that are reasonable for an indoor setting. Figure 5 also highlights the rise in CO_2_ levels of all 3 zones that is a direct result of venting at the time recorded of 2 p.m.

**Figure 6:**
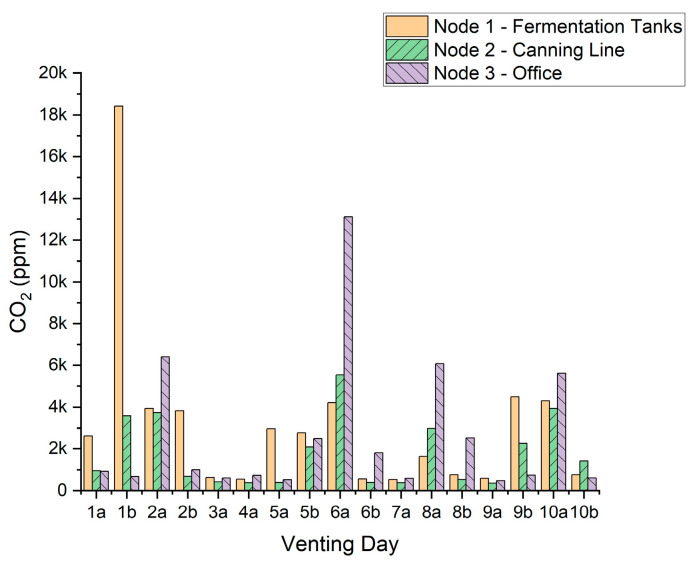
Maximum CO_2_ measured by each sensor node for every venting event.

The maximum CO_2_ concentration (ppm) recorded by each sensor node for each venting event is presented in Figure 6. The maximum is deduced by inspecting the time-dependent CO_2_ concentration for a period of 60 min either side of the nominal vent time, to determine any increase in measured CO_2_ as a result of venting relative to the baseline concentration. In Figure 5, the rise in measured CO_2_ occurs at a similar time following venting. Likewise, the peak measured concentration was reasonably similar at each node. However, this was not always the case and distinct differences both in peak concentration and time to respond varied significantly, without clear or explainable correlations. This is apparent in Figure 6, where there is no obvious relationship between one region of the brewery accumulating CO_2_ more readily than any other. Likewise, this does not appear to be significantly influenced by which tank is vented. In three cases (7, 8a and 10b), no discernible increase in CO_2_ is apparent. However, in the majority of cases, a significant rise in CO_2_ is detectable. One might expect that CO_2_ would accumulate in one area more than others; however, this seems not to be the case when fermentation tanks are vented, irrespective of the position of the tank. Likewise, while the vent durations are almost always 30 min, it is likely that the vented volume of CO_2_ is significantly different in each case, due to differences in flow rate. It would be interesting in future to equip the vessels with flowmeters to record the volume and rate of CO_2_ introduction into the brewery, rather than the time. Equally, there is no indication that longer venting times, e.g., 8a and 10a, lead to higher levels of recorded CO_2_.

While it is perhaps surprising that clearer trends are not apparent, this is likely explained by the complicated air movements within the brewery space, influenced by the movement of people, the use of fans and the presence of a roller door that remains mostly open during working hours, meaning outdoor air flow (wind speed and direction) can play a significant role in how the vented gas moves within the brewery space.

The maximum CO_2_ levels recorded for each node are 18,420 ppm for Node 1 (fermentation tanks), 5538 ppm for Node 2 (the canning line) and 13,100 ppm for Node 3 (the office). It should be noted that the sensor remains accurate to an upper limit of only 10,000 ppm, so values above this are indicative only. That said, the maximum CO_2_ levels are well above the 1000 and 10,000 ppm benchmarks, and it can also be seen from the results that almost every vent, approximately 65% (11/17), have directly influenced excessive CO_2_ levels above 1000 ppm.

It is likely that the observation of the maximum CO_2_ level of 18,420 ppm results from a “perfect storm” of influencing factors. The corresponding venting event took place at 7 p.m., therefore the main source of ventilation (roller door) was not present, as it was shut at roughly 5 p.m. every working day. At the canning line, the CO_2_ peak reaches almost 4000 ppm for the same venting event. For the sensor in the office, a peak is not noticed since the door to the office was also closed at approximately 5 p.m., likely impeding the transfer of air from the main brewery zone. Importantly, it was indicated by the staff that venting after 5 p.m. is not an uncommon occurrence. This leads to the possibility of very high and dangerous levels of CO_2_ being in the brewery space.

Venting event 6a also generates large concentrations of measured CO_2_. This occurs as a result of a refill of CO_2_ storage tanks within the building and is most likely due to vapor from liquid CO_2_ unintentionally venting. Since this process occurs close to the office (Node 3), it is not surprising that Node 3 detects the highest concentration of CO_2_—approximately 13,000 ppm. This again raises a concern for office staff working in the office, as the CO_2_ level remains above 10,000 ppm for 26 min in this case.

**Figure 7:**
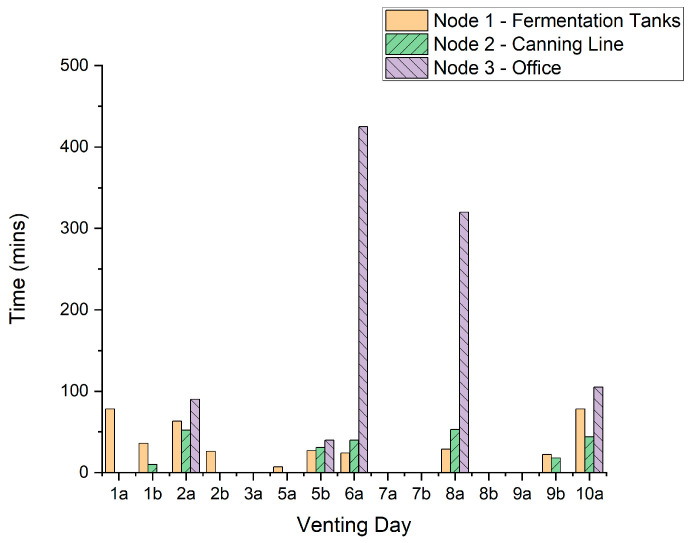
Consecutive time the CO_2_ concentration remained above 1000 ppm for each venting event.

The time (mins) that the CO_2_ level remained above 1000 ppm for each node for each venting occurrence is presented in Figure 7.

The maximum consecutive amount of time CO_2_ levels exceed 1000 ppm for each node is 425 min for Node 3, 78 min for Node 1 and 53 min for Node 2. 10,000 ppm is exceeded in two cases for durations of 26 min for Node 3 and 3 min for Node 1. High CO_2_ levels coupled with the extended periods of potential exposure have the potential to create a hazardous working environment for the brewery staff due to physiological and/or cognitive effects, combined with the nature of work, as discussed in Section 1.2.

For venting events 2a, 6a, 8a, and 10a, where all 3 nodes exceed 1000 ppm, sensor Node 3 measures the longest period for CO_2_ levels above 1000 ppm, indicating that the office area is not well-ventilated compared to other areas of the workplace and strategies should be considered to reduce the excessively long periods (up to 425 min) when CO_2_ exceeds 1000 ppm.

Huizen (2020) [38] measured the CO_2_ level inside several breweries ranging from small (<10,000 bbls) to large (>100,000 bbls), with the current tested brewery being equivalent to a small brewery. Huizen (2020) recorded that the 95% confidence interval of small breweries tested ranged from 2422–2851 ppm. This result reinforces our finding that high concentrations of CO_2_—well above background levels—can be found in breweries. However, it is difficult to directly compare with the results achieved by our system for two reasons. Firstly, Huizen‘s system consisted of a wearable, mobile sensor, not a network of static sensors. Secondly, and perhaps of most significance, our work focuses on the peaks of CO_2_ exposure for periods of CO_2_ venting. Our assumption is that Huizen might also have noted extremely high peaks had the mobile sensors been close to fermentation tanks during indoor exposure, using high sampling frequency.

### 3.2. Beyond a Single Hard-Wired Sensor

The concentration of CO_2_ within the air in the brewery is clearly influenced by the venting of fermentation tanks. However, the identification of differences in measured CO_2_ at different times and locations throughout the brewery (Nodes 1–3) reveals that a single hard-wired CO_2_ sensor may be inadequate to support IAQ monitoring. This strengthens the need to have a network of CO_2_ sensors inside a craft brewery. For this purpose, portable or wearable CO_2_ sensor nodes might be more suitable, imitating the approach taken to radiation dosimetry, for example. This would allow the monitoring of CO_2_ levels for the purpose of protecting the workforce and could safeguard the entire workplace, including inside fermentation tanks where workers often have to place their head, without the need for multiple hard-wired sensors in each area. They could also allow the workers to be warned in real-time of potential CO_2_ dangers that may occur if they proceed with a task.

**Figure 8:**
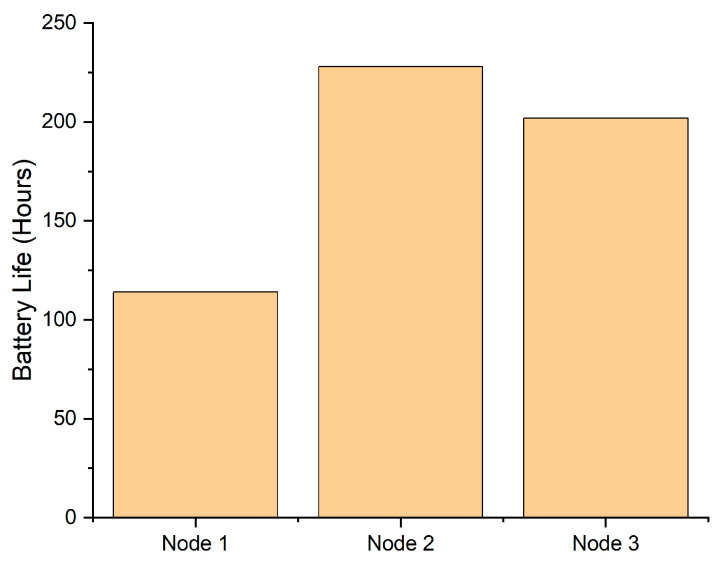
Sensor node battery life.

For portable or wearable sensors the battery life (hours) for each sensor node is a key consideration. While a hard-wired sensor receives constant power, a portable sensor would create a battery replacement/recharging burden. Therefore, a relatively long battery life is required in order for the sensors to be properly used. In Figure 8, the average battery life for the 3 sensor nodes is presented.

The largest battery life was measured for Node 2, at approximately 10 days. However, the battery life of Node 1 was only approximately 4 days. This meant that the batteries for each node had to be changed every 4 days for consistency. The large difference in battery life was mainly attributed to the distance between the sensor node and wireless access point, with larger distances resulting in less battery life due to more power being used to transmit data. This, however, was not the only attributing factor to reduced battery life, as another factor was obstacles in the workplace between the sensor nodes and wireless access point such as piled up cans and kegs.

The extremely short battery life of the current sensor network leaves a lot of room for improvement, and such a short battery life would not be feasible for workplaces adopting IoT technologies [40]. A low-power sensor and communication protocol could be utilized to significantly extend the battery life of sensors [40], making the principle more feasible. This will be investigated in future work. Other strategies to mitigate exposure to CO_2_ in breweries might include careful consideration of ventilation of the vented CO_2_ during brewery design, the possibility of technology that vents the CO_2_ to the outside, personal protective equipment (PPE) for brewery staff, or the enforcement of regular “fresh air” breaks to prevent exposure over significant time frames.

## 4. Conclusions

Maintaining a high standard of indoor air quality (IAQ) is vital to ensuring good human health, since many humans spend much of their time indoors. The measured concentration of CO_2_ in air is a good proxy for IAQ. High levels of CO_2_, above 1000 and 10,000 ppm, have been shown to cause cognitive impairment and physiological impairment, respectively. Work environments that generate CO_2_ as an inherent part of their business present a unique and significant risk in terms of poor IAQ. Craft breweries generate CO_2_ and, unlike larger breweries, often lack the technology to capture and re-use the fermentation CO_2_ for beer carbonation. This study demonstrated that the venting of fermentation CO_2_ and the unintentional venting of CO_2_ during the filling of storage tank can cause the indoor CO_2_ levels to exceed safe limits. This was shown by monitoring CO_2_ levels inside an Australian craft brewery using a system containing three sensor nodes positioned strategically in different sections of the brewery. The maximum CO_2_ level recorded was in excess of 18,000 ppm, with the maximum time period levels exceeded 1000 and 10,000 ppm being equivalent to 425 and 26 min, respectively. The identification of differences in measured CO_2_ at different times and locations throughout the brewery reveals that a single hard-wired CO_2_ sensor may be inadequate to support IAQ monitoring. This strengthens the need to have a network of CO_2_ sensors inside a craft brewery. For this purpose, portable or wearable CO_2_ sensor nodes might be most suitable. The battery life of the sensors is a key consideration, and the current sensor battery life is too short. Low-power sensors and communication protocols are recommended for this task.

## Figures and Tables

**Table 1:** A 10-day subset of CO_2_ venting events within the brewery. Vents occurred for a duration of approximately 30 min, unless otherwise stated.

Venting Day		Vent Time (Duration)	Tank Number
1	a	2 p.m.	6
	b	7 p.m.	BR2
2	a	2 p.m.	4
	b	4 p.m. (Not recorded)	BR1
3	a	2 p.m.	1
4	a	2 p.m.	8
5	a	10 a.m.	9
	b	2 p.m.	Stored CO_2_
6	a	9 a.m.	Stored CO_2_
	b	12 p.m.	3
7	a	11 a.m.	5
8	a	11 a.m. (60 min)	Stored CO_2_
	b	12 p.m.	2
9	a	12 p.m.	7
	b	1 p.m.	10
10	a	9 a.m. (45 min)	Stored CO_2_
	b	11 a.m.	8

## Data Availability

Data from the study is available from the corresponding author by request.
